# Shortened first-line TB treatment in Brazil: potential cost savings for patients and health services

**DOI:** 10.1186/s12913-016-1269-x

**Published:** 2016-01-22

**Authors:** Anete Trajman, Mayara Lisboa Bastos, Marcia Belo, Janaína Calaça, Júlia Gaspar, Alexandre Martins dos Santos, Camila Martins dos Santos, Raquel Trindade Brito, William A. Wells, Frank G. Cobelens, Anna Vassall, Gabriela B. Gomez

**Affiliations:** 1 Federal University of Rio de Janeiro, Rio de Janeiro, Brazil; 2 McGill University, Montreal, Canada; 3 Tuberculosis Scientific League, Rio de Janeiro, Brazil; 4 Souza Marques Foundation, Rio de Janeiro, Brazil; 5 Rio de Janeiro Municipal Health Department, Rio de Janeiro, Brazil; 6 Global Alliance for TB Drug Development, New York, USA; 7 Current address: United States Agency for International Development, Washington, DC USA; 8 Amsterdam Institute for Global Health and Development and Department of Global Health, Academic Medical Center, University of Amsterdam, Amsterdam, The Netherlands; 9 Department of Global Health and Development, London School of Hygiene and Tropical Medicine, London, UK

**Keywords:** Tuberculosis, Antitubercular agents, Brazil, Directly observed therapy, Health care costs

## Abstract

**Background:**

Shortened treatment regimens for tuberculosis are under development to improve treatment outcomes and reduce costs. We estimated potential savings from a societal perspective in Brazil following the introduction of a hypothetical four-month regimen for tuberculosis treatment.

**Methods:**

Data were gathered in ten randomly selected health facilities in Rio de Janeiro. Health service costs were estimated using an ingredient approach. Patient costs were estimated from a questionnaire administered to 126 patients. Costs per visits and per case treated were analysed according to the type of therapy: self-administered treatment (SAT), community- and facility-directly observed treatment (community-DOT, facility-DOT).

**Results:**

During the last 2 months of treatment, the largest savings could be expected for community-DOT; on average USD 17,351-18,203 and USD 43,660-45,856 (bottom-up and top-down estimates) per clinic. Savings to patients could also be expected as the median (interquartile range) patient-related costs during the two last months were USD 108 (13–291), USD 93 (36–239) and USD 11 (7–126), respectively for SAT, facility-DOT and community-DOT.

**Conclusion:**

Introducing a four-month regimen may result in significant cost savings for both the health service and patients, especially the poorest. In particular, a community-DOT strategy, including treatment at home, could maximise health services savings while limiting patient costs. Our cost estimates are likely to be conservative because a 4-month regimen could hypothetically increase the proportion of patients cured by reducing the number of patients defaulting and we did not include the possible cost benefits from the subsequent prevention of costs due to downstream transmission averted and rapid clinical improvement with less side effects in the last two months.

**Electronic supplementary material:**

The online version of this article (doi:10.1186/s12913-016-1269-x) contains supplementary material, which is available to authorized users.

## Background

Globally, in 2013, there were nine million new cases and 1.5 million deaths from tuberculosis (TB), with 95 % of the deaths occurring in low- and middle-income countries [1]. Major advances in diagnostic technologies [2–6] and an increase in treatment coverage [1] have largely contributed to improvements in TB control worldwide. However, the length of the current first-line standard regimen (total of six months, including a 2-month intensive phase with four drugs and a 4-month continuation phase with two drugs), as well as the side effects of this treatment, continue to hamper treatment adherence and acceptability. In addition, treatment costs represent a substantial expenditure for National TB Programmes (NTP) [1, 7], while the costs borne by patients can be disproportionally high considering the poorest subgroups of the population affected by the disease [8, 9]. Thus, substantial investments and international efforts are being put into the development of shortened new regimens, mainly aiming to reduce the length of the continuation phase [10–13].

Brazil has been an early adopter of new technologies in general and of new TB technologies in particular [14, 15]. Accurate information on costs is an important input for planning and budgeting by the Brazilian NTP when considering funding the introduction of new technologies. Introducing a 4-month regimen for TB in a high service cost scenario, such as Brazil, may lead to overall savings from the health service perceptive [16]. However, initial published estimates have been based on global estimates of treatment costs from the World Health Organisation (WHO), rather than locally-collected data and have excluded costs from a patient perspective [16]. Yet, the importance of including patient cost into policy decisions is reflected in and will help track progress of WHO’s post-2035 targets, which include a statement that no household should suffer from catastrophic expenditures related to TB [17]. Previous studies suggest that cost savings from the patient side may be an important advantage of shortened TB regimens. Patient costs during the last two months of first-line treatment were recently shown to correspond to 89 and 77 % of the 2-month average national incomes in Tanzania and Bangladesh [18]. However, to date, there is scarce information about actual resource utilisation, treatment models in place, and how costs related to TB treatment (for patients or the health service) vary depending on the treatment model used. Thus, the purpose of this study is to assess health service and patient costs during the last two months of the current first-line regimen and to explore potential savings following the introduction of a 4-month regimen in Rio de Janeiro, Brazil.

## Methods

### Study setting

The city of Rio de Janeiro has one of the highest TB incidence rates in Brazil [19], with 90 new cases per 100,000 population reported yearly [19]. This high incidence has been linked to structural determinants such as urban violence, poverty, social inequity, and a complex healthcare system [20]. Although, nationally, all TB treatment is available through public facilities free-of-charge and supervised treatment is mandatory, there is important variation among clinics of the treatment models offered. Currently, there are three types of primary care facilities in Rio [21]: (i) family health clinics, where family health teams composed of one physician, one nurse, one aid nurse, and six community healthcare workers provide home visits and clinic-based consultations. Each team is responsible for 1,000 to 1,500 families living in their catchment area; (ii) classic health facilities, where only scheduled appointments with general physicians, specialists, and nurses are available; and (iii) mixed clinics providing both types of services. All facilities provide self-administered therapy (SAT) for TB treatment. However, not all clinics have implemented directly observed therapy (DOT); in those where it has been implemented, two treatment models are available: facility-based and community-based DOT (facility-DOT and community-DOT). Facility-DOT patients have to present to the clinics for pill collection and intake; while community-DOT patients receive daily visits from health care agents distributing pills and supervising intake on working days. The frequency of supervision may also vary across facilities - at least three times weekly, ideally five times, during the first 2 months; twice weekly, ideally five times, for the remaining 4 months of treatment [22] All patients have monthly visits with a physician for clinical, bacteriological and (eventually) radiological evaluation, regardless of the treatment model.

### Data collection and analysis

To ensure a representative range of costs estimates from different settings, we purposively selected four geographical areas to obtain a balance between the municipality of Rio and peri-urban areas. We then randomly sampled 12 facilities from a census of all eligible facilities currently treating at least 50 TB patients (excluding hospitals), stratified by workload (ratio of TB patients per healthcare workers) and type of clinic (family health clinics, classic health facilities, and mixed clinics).

#### Health service costs

Costs from a healthcare provider’s perspective were estimated using an ingredient approach [23–25] for each participating facility. We included capital costs (building, equipment, furniture) and recurrent costs (labour, supplies, utilities, drugs, and monitoring and evaluation). We interviewed personnel, observed consultations and reviewed financial and activity reports to estimate the resource use for each input. In cases where resources were shared between TB and other services, we used the following allocation methods: costs for building space were estimated by multiplying the total building space by the average rental price per square meter, sourced from local rental offices. Building space was then allocated according to service distribution within the clinic’s rooms and time used (from staff interviews and observations). For equipment and furniture, we sourced local market prices for all items and allocated to TB proportionally to the time used and number of patients (from staff interviews and clinic records); staff time dedicated to TB was allocated following observations of practices and staff interviews.

We used a combination of bottom-up and top-down approaches to calculate unit costs. In a bottom-up approach, we identified the resources used within a certain input type and after allocating a value to them, we added them to calculate unit costs; for the top-down approach, we divided the total expenditure for a given input type by the units of activity. We adopted a micro-costing approach that mixed both methods. Drug costs are only reported as bottom-up estimates because records for drug expenditures were not reliable at facility-level. In Brazil, drug expenditure is centralized; and therefore not accessible at the facility level. We verified the prices of drugs and consumables sourced from suppliers with international databases [26]. Capital costs were annualized and discounted at 3 % per year. All prices were collected in local currency.

We calculated cost per visit and episode costs (cost per person treated) by treatment modality and site. We extracted information on the total number of non-multidrug resistant TB patients being treated for each treatment modality (SAT, facility- and community-DOT) from service statistics for the period covered. We estimated the episode costs by month by multiplying the number of visits in this period by the unit costs. To allocate costs to the last two months of treatment, we first estimated the number of visits using two methods: (i) assuming a 10 % default rate after the 4th month, we estimated that the number of monthly visits would be 45 % of the number of visits if guidelines were fully adhered to; (ii) we estimated the number of visits during the last two months as reported by patients in the questionnaires. We did not include programme costs, defined as the costs associated with general support to service delivery (i.e., not directly linked to individual patients; for example administration, management, and supervision activities).

Finally, we estimated the total potential annual savings due to the introduction of a shortened first-line TB regimen considering that 76,020 persons were treated with TB in 2013 [1] and assuming a distribution of treatment models as seen in our participating clinics.

#### Patient costs

All TB patients attending (or having home visits by a community health worker) at a participating clinic on their 5th or 6th month of treatment were invited to participate. After obtaining informed consent, data on demographics, assets, type of TB treatment and both direct and indirect costs were gathered. The questionnaire used to collect patient-related costs was based on a validated tool for patient costs developed by USAID and available online [27]. Direct costs included all out-of-pocket expenditure, while indirect costs included productivity loss/opportunity cost to the household, coping costs (selling of assets and borrowed money) and carer’s costs. With regards to productivity loss/opportunity costs to the household, we considered both transport time seeking care and additional days off-work due to the disease. The value of an hour was estimated using four different methods: (i) 2013 minimum wage; (ii) midpoint of the estimated family monthly income using *Critério Brasil* [28] divided by the number of persons living in the house; (iii) the shadow salary [29], estimated using the union’s hourly salary, according to the profession declared (for those without employment, we valued their hourly cost based on the minimal wage); and (iv) the declared income. We assumed a 44-hour weekly contract for participants employed [30].

Costs per visit were multiplied by the number of visits in the last 2 months to arrive at total costs. We calculated the ratio of total costs during the last 2 months of treatment to the estimated monthly income by estimation method. We considered total costs during the last 2 months to be catastrophic when they represented over 40 % of the monthly income [31].

Data collection took place from June 2013 to May 2014. The average exchange rate used was USD 1 = BRL 2.16. All health service cost analyses were performed using EXCEL 2013 (Microsoft, USA). Statistics analyses were performed in SPSS 21 (IBM, USA). Mean costs and standard deviations (SD) were compared using independent *t*-test.

### Ethical approval

The study was approved by the Institutional Review Board at the *Universidade Gama Filho* (268.778) in Rio de Janeiro; the *Comitê de Ética em Pesquisa, Prefeitura do Rio de Janeiro* (164A/2013); and the Observational and Interventions Research Ethics Committee of the London School of Hygiene and Tropical Medicine, UK (6322).

## Results

The 12 randomly selected clinics provided services to neighbourhoods with a wide range of income (average monthly income USD 209-USD 2,538) and TB burden (incidence rate 51.2–382.4 new TB cases per 100,000 population per year). All facilities offered SAT and at least one type of DOT (9 offered facility- DOT while the other three offered community-DOT). The facilities presented a wide workload range (50–383 staff registered; 66–332 patients per year). Of 12 clinics approached, ten (83.3 %) agreed to participate (Table 1).

**Table 1:** Characteristics of all randomly selected clinics

Study Information		Clinic information				Neighbourhood information		
Clinic	Included	n FTE staff	n TB patients/year	Type [43]	TB treatment model	Population (2010) [44]	TB incidence rate^a^ [45]	Average monthly income (2013USD) [44]
1	No	137 [43]	76 [43]	Family health clinic	SAT and community-DOT	10,086	152.2	2,538.1
2	Yes	56	51	Family health clinic	SAT and community-DOT	7,390	51.2	610.3
3	Yes	147	163	Family health clinic	SAT and community-DOT	58,007	382.4	208.8
4	Yes	89	117	Mixed clinic	SAT and facility-DOT	23,861	220.0	401.5
5	Yes	138.5	66	Mixed clinic	SAT and facility-DOT	133,315	108.1	356.0
6	Yes	173.5	98	Classic health facility	SAT and facility-DOT	286,527	79.7	387.8
7	Yes	268	26	Classic health facility	SAT and facility-DOT	182,722	109.4	249.2
8	Yes	111	132	Mixed clinic	SAT and facility-DOT	137,710	103.0	1,253.6
9	Yes	188	195	Mixed clinic	SAT and facility-DOT	41,524	112.8	357.5
10	Yes	76.5	107	Mixed clinic	SAT and facility-DOT	14,794	95.6	1,835.7
11	Yes	256	178	Classic health facility	SAT and facility-DOT	211,068	167.2	315.6
12	No	201 [43]	254 [43]	Mixed clinic	SAT and facility-DOT	33,180	56.8	480.8

^a^per 100,000 inhabitants, in 2009; *n* number; *FTE* full-time equivalent; *SAT* self-administered therapy; *DOT* directly observed therapy

### Health service costs

Service utilisation for TB is presented by facility in Additional file 1: Table S1 in the supplementary material. A total of 1,140 TB patients were treated during 2013. Of these, seven were multidrug-resistant TB patients (0.6 %) treated in one facility only. These patients were not included in the analysis. Of the 1,133 new or previously treated TB patients, 739 (65 %) were DOT patients (530, 47 % in facility-DOT and 209, 18 % in community-DOT). The total number of visits was estimated to be 55,876, of which 37 % (*n* = 20,691) were community visits. Only 4 % of all visits (*n* = 2,325) were SAT visits.

The unit and total costs by treatment type as well as drug and episode costs by treatment phase and type are presented in Additional file 1: Table S2 in supplementary material. Detailed unit costs by cost input are displayed in Additional file 1: Table S3, also in supplementary material. Briefly, the highest mean unit cost excluding drugs was estimated for SAT at USD 31.1 (range 8.0–93.1) and USD 61.1 (range 18.3–264.4); the estimates for facility-DOT were USD 8.2 (range 3.9–19.7) and USD 14.8 (range 5.3–21.7); while for community-DOT, these were USD 5.4 (range 5.2–5.7) and USD 14.5 (range 13.0–16.0), bottom-up and top-down respectively. The highest mean cost per patient completing treatment was observed for community-DOT (USD 571.1 and USD 1,477, bottom-up and top-down respectively) but varied greatly among sites (ranges 549.2–594.1 and 1,331–1,623). Mean total annual costs per facility were estimated at USD 43,439 and 81,465, bottom-up and top-down respectively. Staff and building costs represented the main type of costs in both SAT and facility- DOT, whereas for community- DOT, the main driver of costs was staff and overhead costs (Additional file 1: Table S3).

Table 2 presents the mean potential cost savings in the last two months of treatment by utilisation assumption (see Additional file 1: Table S4 for top-down estimates and Additional file 1: Table S5 for bottom-up estimates by site). Our estimated savings varied by clinic and type of treatment. The largest savings could be expected for community-DOT, reaching for example, up to USD 67,226 (Additional file 1: Table S4) at a single clinic. We estimated that the national introduction of a shortened regimen could potentially save up to USD 8,345,775 and 16,166,046 (top-down and bottom-up estimates) savings in treatment costs related to the two last months of treatment.

**Table 2:** Potential savings: episode and total costs by type of treatment in the last two months for different utilisation assumptions, USD 2013

	Top-down; mean (range)	Bottom-up; mean (range)
EPISODE COSTS		
Facility-DOT		
45 % continuation phase	283.7 (108.5–410.5)	160.6 (82.7–373.3)
Patient utilisation	286.9 (109.6–415.2)	162.3 (83.5–377.6)
SAT		
45 % continuation phase	115.1 (38.9–470.8)	61.6 (20.8–168.5)
Patient utilisation	127.7 (42.6–525.0)	68.0 (22.4–187.6)
Community-DOT		
45 % continuation phase	441.5 (397.6–485.6)	169.8 (162.8–176.8)
Patient utilisation	463.8 (417.6–510.1)	178.1 (170.8–185.6)
Average total costs by treatment type		
Facility-DOT		
45 % continuation phase	16,834 (2,824–24,631)	10,083 (848–18,260)
Patient utilisation	17,024 (2,855–24,911)	10,194 (857–18,467)
SAT		
45 % continuation phase	2,021 (65.6–4,701)	1,287 (55.4–3,562)
Patient utilisation	2,227 (72.5–5,157)	1,407 (61.1–3,884)
Community-DOT		
45 % continuation phase	43,660 (23,308–64,012)	17,351 (8,486–26,216)
Patient utilisation	45,856 (24,486–67,226)	18,203 (8,907–27,500)
Average total costs per facility		
45 % continuation phase	24,221 (4,010–64,954)	12,824 (1,316–26,551)
Patient utilisation	25,018 (4,159–68,276)	13,204 (1358–27873)

*DOT* directly observed therapy; *SAT* self-administered therapy

Utilisation assumptions: 1) 45 % continuation phase. In this scenario we calculated the costs in the last two months of treatment as a proportion of the continuation phase costs; 2) Patient utilisation. In this alternative scenario, costs in the last two months of treatment are calculated using the reported number of visits by patients interviewed

### Patient costs

We interviewed a total of 126 patients in the ten participating clinics. The median age among our sample was 43.0 (interquartile range 27.8–55.4) years, 61 % (*n* = 77) were male, 78 % (*n* = 93) belonged to the three lowest socioeconomic classes. Participants’ characteristics are detailed in Additional file 1: Table S6 in supplementary material.

The estimated value of a productive hour using the different methods was: (i) minimum wage, USD 1.4/h; (ii) family monthly income per capita, USD 1.0 (IQR 0.7–1.8)/h; (iii) shadow price, USD 2.1 (IQR 2.0–2.2)/h; and (iv) declared income, USD 1.9 (IQR 1.5–3.2)/h. Among patients declaring their income (*n* = 49), the median declared monthly income was USD 370 (IQR 298–625). Using the shadow salary method, the median monthly income was USD 420 (IQR 405–435); while using the family income per capita, the median monthly income was estimated at USD 195 (IQR 133–359).

Table 3 shows direct costs as well as indirect by method to value a productive hour. Mean direct costs during the last 2 months were USD 96 for the whole sample. The mean total indirect costs varied depending on the method used to value a productive hour: using the minimum wage and the SES income per capita, the mean total indirect costs was USD 316; using the income per activity, this was USD 332 for the whole sample. If we restrict the sample to those reporting an income, then the mean total indirect costs is estimated at USD 357.

**Table 3:** Total, direct and indirect costs incurred by patients during the last two months of treatment in Rio de Janeiro, Brazil (using four methods for productivity loss valuation^a^), USD 2013

Costs	Minimum wage, mean (SD) *N* = 126	SES income per capita, mean (SD) *N* = 126	Income per activity, mean (SD) *N* = 126	Reported income, mean (SD) *N* = 49
Total direct costs (all)^b^	96 (148)			114 (162)
Transport	8 (19)			5 (11)
Food	2 (5)			2 (5)
Other non-transport/food	3 (21)			0.5 (3)
Nutritional supplements	82 (143)			105 (158)
Total indirect costs (all)^b^	316 (1,466)	316 (1,469)	332 (1,472)	357 (1,985)
Coping costs^d^	275 (1,458)			327 (1,982)
Productivity loss	21 (24)	18 (26)	30 (32)	25 (39)
Caregiver/guardian costs	20 (67)	22 (78)	27 (94)	5 (23)
Total costs (all)^b^	413 (1,503)	413 (1,507)	153 (206)	144 (163)
Total costs (patients only)^c^	361 (1,481)	364 (1,484)	374 (1,486)	411 (1,991)
Total direct costs	51 (74)			57 (77)
Total indirect costs	310 (1,466)	313 (1,469)	323 (1,471)	354 (1,981)

^a^Minimum wage refers to the valuation of an productive hour lost based on the minimum wage in Brazil and a 44 weekly hour contract; SES income per capita refers to the valuation of a productive hour based on the average income for a reported socio-economic status; Income activity refers to the valuation of a productive hour based on the average income for the patient’s profession, based on union’s information; Reported income refers to the valuation of a productive hour based on the income reported by the participant. ^b^These costs are presented for both patients and any person accompanying the patient during their visits. ^c^These costs refer to costs incurred by patients only, not including costs incurred by any accompanying person. ^d^Coping costs include borrowed money and selling of assets

When comparing the mean total costs by participant’s characteristics, these were higher for patients belonging to lower socioeconomic classes (mainly due to higher indirect costs), and patients under SAT and facility-DOT regimens (Table 4). However, while the difference was statistically significant when comparing socioeconomic classes, it was not statistically significant when comparing type of treatment. Total costs during the last two months of treatment corresponded to a maximum of 26.3 % (1.6–41.8 %) if minimum wage method was considered and a minimum of 10.4 % (1.4–41.8 %) when income per capita for the socio-economic class was considered. These proportions of the income attaining over 40 % for 56 (44 %) patients and 34 (27 %) respectively (Additional file 1: Table S7 in supplementary material).

**Table 4:** Factors associated with costs incurred by patients during the last two months of treatment in Rio de Janeiro, using income per activity reported by participant to value a productive hour (USD 2013)

Variables	Number in each category with direct costs	Direct costs, mean (SD) *N* = 85^b^	*P* value^a^	Indirect costs, mean (SD) *N* = 126	*P* value^a^	Total costs, mean (SD) *N* = 126	*P*-value^a^
Sex^b^							
Female	32	79 (126)	0.313	355 (1,981)	0.892	127 (145)	0.263
Male	53	107 (161)		318 (1,040)		169 (236)	
Age							
< 43 years	41	105 (165)	0.527	289 (998)	0.743	167 (251)	0.451
> = 43 years	44	88 (131)		375 (1834)		139 (139)	
Treatment history							
New case	71	101 (155)	0.544	373 (1,628)	0.524	153 (217)	0.952
Retreatment	14	80 (116)		159 (299)		150 (157)	
Residence							
Urban	56	88 (152)	0.351	258 (978)	0.386	135 (213)	0.166
Peri-urban	29	114 (140)		500 (2,193)		190 (187)	
Treatment type							
SAT	52	110 (165)	0.379	560 (49)	0.216	170 (256)	0.333
Facility-DOT	29	93 (137)		126 (185)		154 (142)	
Community-DOT	4	52 (104)		39 (49)		81 (122)	
Socioeconomic status^c^							
A/B (median family income USD 4288.4-USD 1228.7)	18	60 (108)	0.128	68 (137)	**0.042**	98 (121)	0.099
C/D/E (median family income USD 780.1-USD 359.2)	64	111 (160)		428 (1,703)		175 (227)	

*Abbreviations*: *SAT* self-administered therapy; *DOT* directly observed therapy; *SD* standard deviation

^*^from independent *t*-test

^b^Among 126 patients, 85 patients or accompanying persons declared direct costs

^c^missing information for 3 participants

Bold numbers highlight significant *p*-values.

## Discussion

We present a comprehensive cost study describing detailed information on first-line regimen costs that can serve as an input for decision-making and budget preparation for NTPs when considering the introduction of new shortened regimens. We estimated the methods of delivery to be the main driver of overall costs. From a health service perspective, community-DOT was the main contributor to total costs for first-line TB treatment in these primary care facilities, mainly due to high service utilisation (domiciliary visits by community health care agents). SAT was estimated to be the treatment modality with the highest unit cost, even though it was the treatment model contributing the least to total costs, due to the least number of visits. From a patient perspective, facility-based services (both SAT and facility-DOT) were modalities representing the highest costs for the patients, mainly associated to transport and productivity loss costs. Therefore, while bringing services to the community might seem more costly from a health service perspective, it can lead to substantial savings from a societal perspective. Besides costs, decision-makers should take into account the potential improvement brought by community-based treatment regarding treatment outcomes and equity in the access to care for an already vulnerable population. While the patient-level benefits of community-DOT compared to facility-DOT have been suggested in three clinical trials, the benefits of DOT over SAT are still controversial [32, 33]. Additionally, although general access to care and healthcare utilization has improved with the Family Health Program expansion in Brazil [34], TB treatment outcomes have not [35].

In terms of cost differences among models of service delivery, our results are consistent with two previous studies in the country [7, 36]. However, Steffen et al. estimates were based on staff costs exclusively and the authors assumed that service utilisation at the facility-level was the same for SAT or DOT, but much lower for pill collection visits, explaining the lower cost estimates for the pill collecting visits at USD 7.5 (USD 9.4 in 2012) [7]. In addition, at the time of study (2008), community-DOT was the exception in Rio de Janeiro. Although they found SAT to be more cost-effective than DOT, community-DOT was not evaluated. In our study, community-DOT was the strategy with the lowest unit costs, both from the health service and patient perspectives.

We found a large variation both across clinics and between the top-down and bottom-up approaches, mainly due to estimations of staff time. While a top-down method might be less precise when disaggregating costs per activity, especially for staff costs allocation, it might be a more accurate representation of total costs, including inefficiencies in service delivery. Compared to the USD 88,000,000 national budget for TB control, the potential estimated savings should a four-month regimen be introduced nationally could represent up to 9–18 % of the NTP budget. These savings could be realised in their totality if, for example, staff would be relocated to other programmes. As this might not be the case, we are offering a top end estimate of potential savings. On the other hand, our estimates of potential cost benefits are likely to be conservative because potential savings due to a possible reduction in transmission and the possible differential in health improvements, with less side effects and therefore service utilisation during the last months of treatment, were not considered.

Costs of treatment for patients can be a significant barrier to access and utilisation of healthcare [18, 37–39], even in health systems where drugs and services are free-of-charge, as is the case in Brazil. In the present study, during the two last months of treatment, when patients have usually recovered from symptoms present at diagnosis and are often fit to work, the costs incurred by patients still represent a substantial proportion of their income. We estimated that the introduction of shortened regimens may prevent catastrophic expenditure for 27 to 44 % of individuals in our sample, depending on the method used to estimate the value of a productive hour. This result is comparable to previous results in Tanzania and Bangladesh, where the total patients costs of six months of treatment represented 103 and 117 % of the per capita income, respectively [18].

In our study, costs for patients were significantly higher for those in the lower socioeconomic classes. Because their productive hour is less valued, we would expect lower indirect costs. Our finding (higher indirect costs) suggests that, no matter which method is used to estimate them, more hours are spent in or traveling to the facilities and raises more concern about the added vulnerability to an already disadvantaged population. The introduction of a shortened regimen would then benefit lower socio-economic class patients the most.

Our study presents several limitations. We collected data in one only city, including both urban and peri-urban but excluding rural settings. Therefore, the findings can only be generalised to similar settings within Brazil. Additionally, we present potential cost savings during the last 2 months of treatment only and patients incur substantial costs during the pre-diagnosis period [40–42]. In our analysis, we assumed that these costs (pre-diagnosis) would not change, should a shortened regimen be introduced. Additionally, while we aimed to report income loss, this measure might not represent societal productivity loss (due to replacement). An additional limitation is the use of routine data to measure service utilisation. Program indicators reported patients by treatment delivery model but not by treatment phase. The assumptions made about the distribution between patients in the intensive as opposed to continuation phase may have led to an over-estimation of unit costs. Conversely, the study presents significant strengths. Detailed cost data were collected from a societal perspective, from patients and clinics in diverse neighbourhoods adopting different models of treatment. Finally, when faced with uncertainty in assumptions such as the value of a productive hour and the distribution of patients between intensive and continuation phase, we present several estimation methods to test the robustness of our conclusions to these assumptions.

## Conclusion

Altogether, our findings show that significant savings can be achieved for patients and the health system with the introduction of shortened regimens. This is an important finding that sustains the rationale for shorter regimens even with equivalent effectiveness to the currently recommended 6-month regimen. We also highlighted the importance of considering the variation among models of delivery when undertaking cost-effectiveness analyses of future drugs. As the numbers of confirmed TB patients may increase with the introduction of new diagnostics [14], potentially reducing the number of patients empirically treated, it will be important to continue investing on TB programs, not only due to the potential health impact on patients outcomes but also to achieve potential cost gains, helping to avoid catastrophic costs for patients and to attain the post-2015 targets.

## Additional file

Additional file 1:
**Supplement material.** (DOCX 69 kb)

## Abbreviations

BRL: Brazilian real
DOT: directly observed treatment
IQR: interquartile range
NTP: National Tuberculosis Programme
SAT: self-administered treatment
TB: tuberculosis
USD: United States dollar
WHO: World Health Organization
