# Supplementary material for: Shortened first-line TB treatment in Brazil: potential cost savings for patients and health services
Source: BMC Health Serv Res. 2016 Jan 22;16:27. doi: 10.1186/s12913-016-1269-x (PMC4722708; doi:10.1186/s12913-016-1269-x)
Supplement: Additional file 1: — Supplement material. (DOCX 69 kb) [file 12913_2016_1269_MOESM1_ESM.docx]

**Additional material**

Additional results for “Shortened first-line TB treatment in Brazil: potential cost savings for patients and health services. Anete Trajman et al.”

Contents

[**Table S1 –** TB service utilisation by site. 2](#_Toc433238840)

[**Table S2 –** Unit, episode and total costs by site and type of treatment (USD 2013). 3](#_Toc433238841)

[**Table S3 –** Unit costs, excluding drugs, by input type and site (USD 2013). 5](#_Toc433238842)

[**Table S4 –** Potential savings: episode and total costs by type of treatment in the last two months of treatment for different utilisation assumptions (bottom-up estimates by site, USD 2013). 6](#_Toc433238843)

[**Table S5 –** Potential savings: episode and total costs by type of treatment in the last two months of treatment for different utilisation assumptions (top-down estimates by site, USD 2013). 7](#_Toc433238844)

[**Table S6 –** Demographic and clinical characteristics of TB patients interviewed during the last two months of first-line treatment in Rio de Janeiro, Brazil. 8](#_Toc433238845)

[**Table S7** - Proportion of total costs over income, according to different methods to estimate income. 9](#_Toc433238846)

[References 10](#_Toc433238847)

## **Table S1 –** TB service utilisation by site.

| **Clinic number** | **2** | **3** | **4** | **5** | **6** | **7** | **8** | **9** | **10** | **11** |
| --- | --- | --- | --- | --- | --- | --- | --- | --- | --- | --- |
| Total number of non-MDR TB patients | 51 | 163 | 117 | 66 | 98 | 26 | 132 | 195 | 107 | 178 |
| Number of new TB patients | n/a | 139 | 109 | 64 | 74 | 22 | 115 | 185 | 94 | 149 |
| Number of new TB patients, HIV positive | n/a | 7 | 21 | 6 | 14 | 12 | 20 | 10 | 9 | 10 |
| Number of previously treated TB patients | n/a | 24 | 8 | 2 | 24 | 4 | 17 | 10 | 13 | 29 |
| Number of previously treated TB patients, HIV positive | n/a | 3 | 2 | - | 5 | 3 | 5 | 1 | 2 | 7 |
| Number of MDR TB patients | n/a | - | - | - | - | - | - | 7 | - | - |
| Number of MDR TB patients, HIV positive | n/a | - | - | - | - | - | - | 1 | - | - |
| Number of non-MDR TB patients in facility-DOT (%) | n/a | n/a | 109 (93.2) | 65 (98.5) | 54 (55.1) | 8 (30.8) | 81 (61.4) | 74 (37.9) | 43 (40.2) | 96 (53.9) |
| Number of non-MDR TB patients in SAT (%) | 3 (5.9) | 2 (1.2) | 8 (6.8) | 1 (1.5) | 44 (44.9) | 18 (69.2) | 51 (38.6) | 121 (62.1) | 64 (59.8) | 82 (46.1) |
| Number of non-MDR TB patients in community-DOT (%) | 48 (94.1) | 161 (98.8) | n/a | n/a | n/a | n/a | n/a | n/a | n/a | n/a |
| Number of visits per year (%), if facility-DOT | n/a | n/a | 6,758 (99.3) | 4,030 (99.9) | 3,348 (92.8) | 496 (82.4) | 5,022 (94.3) | 4,588 (86.5) | 2,666 (87.6) | 5,952 (92.5) |
| Number of visits per year (%), if SAT | 18 (0.4) | 12 (0.1) | 47 (0.7) | 6 (0.1) | 260 (7.2) | 106 (17.6) | 301 (5.7) | 714 (13.5) | 378 (12.4) | 484 (7.5) |
| Number of visits per year (%), if community-DOT | 4,752 (99.6) | 15,939 (99.9) | n/a | n/a | n/a | n/a | n/a | n/a | n/a | n/a |
| Total number of visits per year for non-MDR TB treatment | 4,770 | 15,951 | 6,805 | 4,036 | 3,608 | 602 | 5,323 | 5,302 | 3,044 | 6,436 |

MDR, multi-drug resistance; DOT, directly observed therapy; SAT, self-administered therapy; n/a, not applicable or not available.

## **Table S2 –** Unit, episode and total costs by site and type of treatment (USD 2013).

| **Clinic number** | **2** | | **3** | | **4** | | **5** | | **6** | | **7** | |
| --- | --- | --- | --- | --- | --- | --- | --- | --- | --- | --- | --- | --- |
|  | **TD** | **BU** | **TD** | **BU** | **TD** | **BU** | **TD** | **BU** | **TD** | **BU** | **TD** | **BU** |
| UNIT COSTS (per visit, excluding drugs) | | | | | | | | | | |  | |
| Facility-DOT | - | - | - | - | 5.3 | 3.9 | 10.7 | 4.9 | 17.3 | 4.6 | 18.5 | 5.2 |
| SAT | 125.4 | 93.2 | 264.5 | 91.6 | 39.8 | 18.4 | 33.6 | 27.8 | 30.5 | 8.1 | 31.9 | 9.1 |
| Community-DOT | 16.0 | 5.7 | 13.0 | 5.2 | - | - | - | - | - | - | - | - |
| DRUG COSTS (per phase)^1^ | | | | | | | | | | |  | |
| Intensive phase | - | 11.5 | - | 15.3 | - | 15.3 | - | 15.3 | - | 15.3 | - | 15.3 |
| Continuation phase | - | 9.9 | - | 13.3 | - | 19.9 | - | 13.3 | - | 13.3 | - | 19.9 |
| EPISODE COSTS (per patient, excluding drugs)^2^ | | | | | | | | | | |  | |
| Facility-DOT (total number of visits: 20.7 in the intensive phase and 41.3 in the continuation phase) | | | | | | | | | | |  | |
| Intensive phase | - | - | - | - | 109.7 | 80.9 | 221.2 | 101.8 | 358.3 | 94.3 | 382.1 | 107.0 |
| Continuation phase | - | - | - | - | 218.9 | 161.5 | 441.4 | 203.1 | 714.9 | 188.2 | 762.3 | 213.5 |
| per patient | - | - | - | - | 363.8 | 277.7 | 691.2 | 333.4 | 1,101.9 | 311.1 | 1,179.6 | 355.8 |
| SAT (total number of visits: 2 in the intensive phase and 3.9 in the continuation phase) | | | | | | | | | | |  | |
| Intensive phase | 250.7 | 186.3 | 528.9 | 183.2 | 79.5 | 36.7 | 67.2 | 55.5 | 60.9 | 16.1 | 63.7 | 18.2 |
| Continuation phase | 488.9 | 363.3 | 1,031.4 | 357.3 | 155.0 | 71.6 | 131.0 | 108.3 | 118.7 | 31.4 | 124.3 | 35.6 |
| per patient | 761.1 | 571.1 | 1,588.9 | 569.1 | 269.8 | 143.6 | 226.8 | 192.5 | 208.2 | 76.1 | 223.3 | 89.1 |
| Community-DOT (total number of visits: 33.3 in the intensive phase and 66.6 in the continuation phase ) | | | | | | | | | | |  | |
| Intensive phase | 534.0 | 190.9 | 434.4 | 173.5 | - | - | - | - | - | - | - | - |
| Continuation phase | 1,068.0 | 381.8 | 868.8 | 347.1 | - | - | - | - | - | - | - | - |
| per patient | 1,623.5 | 594.1 | 1,331.7 | 549.2 | - | - | - | - | - | - | - | - |
| TOTAL COSTS PER FACILITY (by treatment type)^3^ | | | | | | | | | | |  | |
| Facility-DOT | - | - | - | - | 39,663 | 30,278 | 37,328 | 18,008 | 59,503 | 16,801 | 9,437 | 2,846 |
| SAT | 2,283 | 1,713 | 3,177 | 1,138 | 2,158 | 1,149 | 9,979 | 8,470 | 9,164 | 3,349 | 4,020 | 1,603 |
| Community-DOT | 77,928 | 28,520 | 214,417 | 88,430 | - | - | - | - | - | - | - | - |
| TOTAL COSTS^4^ | 80,211 | 30,233 | 217,595 | 89,568 | 41,821 | 31,427 | 45,159 | 21,868 | 68,667 | 20,150 | 13,457 | 4,450 |

**Table S2** (continuation)

| **Clinic number** | **8** | | **9** | | **10** | | **11** | | **TD** | | **BU** |
| --- | --- | --- | --- | --- | --- | --- | --- | --- | --- | --- | --- |
|  | **TD** | **BU** | **TD** | **BU** | **TD** | **BU** | **TD** | **BU** | **Mean (range)** | | **Mean (range)** |
| UNIT COSTS (per visit, excluding drugs) | | | | | | | | | | |  |
| Facility-DOT | 15.2 | 11.9 | 17.6 | 10.4 | 21.7 | 19.7 | 12.7 | 5.5 | 14.8 (5.3-21.7) | | 8.2 (3.9-19.7) |
| SAT | 28.3 | 21.8 | 18.4 | 13.0 | 23.0 | 19.6 | 21.1 | 9.2 | 61.6 (18.4-264.4) | | 31.1 (8.1-93.2) |
| Community-DOT | - | - | - | - | - | - | - | - | 14.5 (13.0-16.0) | | 5.4 (5.2-5.7) |
| DRUG COSTS (per phase)^1^ | | | | | | | | | |  | |
| Intensive phase | - | 11.5 | - | 15.3 | - | 15.3 | - | 15.3 | - | | 14.5 (11.4-15.3) |
| Continuation phase | - | 9.9 | - | 13.3 | - | 13.3 | - | 13.3 | - | | 13.9 (9.9-19.8) |
| EPISODE COSTS^2^ | | | | | | | | | |  | |
| Facility-DOT (total number of visits: 20.7 in the intensive phase and 41.3 in the continuation phase) | | | | | | | | | |  | |
| Intensive phase | 315.3 | 245.5 | 363.3 | 214.5 | 449.8 | 408.4 | 263.3 | 113.8 | 307.9 (119.7-449.8) | | 170.8 (80.9-408.4) |
| Continuation phase | 629.2 | 489.9 | 724.9 | 428.0 | 897.5 | 814.8 | 525.3 | 227.1 | 614.3 (218.9-897.5) | | 340.8 (161.5-814.8) |
| per patient | 966.0 | 756.9 | 1,116.8 | 671.1 | 1,376 | 1,251.8 | 817.2 | 369.5 | 951.6 (363.8-1,376) | | 540.9 (277.7-1,251) |
| SAT (total number of visits: 2 in the intensive phase and 3.9 in the continuation phase) | | | | | | | | | |  | |
| Intensive phase | 56.5 | 43.5 | 36.7 | 25.9 | 46.0 | 39.1 | 42.1 | 18.4 | 123.2 (36.7-528.9) | | 62.3 (16.1-186.3) |
| Continuation phase | 110.1 | 84.9 | 71.6 | 50.6 | 89.7 | 76.2 | 82.1 | 36.0 | 240.3 (71.6-1,031) | | 121.5 (31.4-363.3) |
| per patient | 188.1 | 149.8 | 136.9 | 105.2 | 164.3 | 143.9 | 152.8 | 83.0 | 392.0 (136.9-1,588) | | 212.3 (76.1-571.1) |
| Community-DOT (total number of visits: 33.3 in the intensive phase and 66.6 in the continuation phase ) | | | | | | | | | |  | |
| Intensive phase | - | - | - | - | - | - | - | - | 484.2 (434.4-534.0) | | 182.2 (173.5-190.9) |
| Continuation phase | - | - | - | - | - | - | - | - | 968.4 (868.8-1,068) | | 364.4 (347.1-381.8) |
| per patient | - | - | - | - | - | - | - | - | 1,477 (1,331-1,623) | | 571.7 (549.2-594.1) |
| TOTAL COSTS PER FACILITY (by treatment type)^3^ | | | | | | | | | |  | |
| Facility-DOT | 78,250 | 61,309 | 48,026 | 28,860 | 59,169 | 53,828 | 78,458 | 35,478 | 56,508 (9,437-78,458) | | 33,985 (2,846-61,309) |
| SAT | 9,594 | 7,643 | 8,762 | 6,736 | 10,520 | 9,214 | 12,531 | 6,812 | 7,024 (2,158-12,531) | | 4,555 (1,138-9,214) |
| Community-DOT | - | - | - | - | - | - | - | - | 146,172 (77,928-214,417) | | 58,475 (28,520-88,430) |
| TOTAL COSTS^4^ | 87,844 | 68,952 | 99,217 | 62,403 | 69,689 | 63,042 | 90,990 | 42,291 | 81,465 (13,457-217,595) | | 43,439 (4,450-89,568) |

TD, top-down; BU, bottom-up; DOT, directly observed therapy; SAT, self-administered therapy. ^1^Drug costs are only reported as bottom-up estimates because records for drug expenditures were not reliable at facility-level. ^2^Episode costs: intensive and continuation phase costs are presented excluding drug costs (calculated as the unit cost per visit excluding drug costs*the number of visits in each phase). Total episode cost per patient includes intensive and continuation phase episode costs excluding drug costs + total drug costs. ^3^Total costs per facility by treatment modality were calculated as the total episode cost per patient including drugs*number of patients in each treatment modality during the year.^4^Total annual costs per facility are the sum of total costs by treatment modality.

## **Table S3 –** Unit costs, excluding drugs, by input type and site (USD 2013).

| **Clinic number** | **2** | | **3** | | **4** | | **5** | | **6** | | **7** | | **8** | | **9** | | **10** | | **11** | |
| --- | --- | --- | --- | --- | --- | --- | --- | --- | --- | --- | --- | --- | --- | --- | --- | --- | --- | --- | --- | --- |
|  | **TD** | **BU** | **TD** | **BU** | **TD** | **BU** | **TD** | **BU** | **TD** | **BU** | **TD** | **BU** | **TD** | **BU** | **TD** | **BU** | **TD** | **BU** | **TD** | **BU** |
| *Facility-DOT* | *-* | *-* | *-* | *-* | 5.3 | 3.9 | 10.7 | 4.9 | 17.3 | 4.6 | 18.5 | 5.2 | 15.2 | 11.9 | 17.6 | 10.4 | 21.7 | 19.7 | 12.7 | 5.5 |
| Building |  |  |  |  | 1.6 | 1.6 | 1.6 | 1.6 | 2.6 | 2.6 | 1.6 | 1.6 | 7.5 | 7.5 | 5.5 | 5.5 | 14.7 | 14.7 | 2.1 | 2.1 |
| Equipment |  |  |  |  | 0.0 | 0.0 | 0.0 | 0.0 | 0.1 | 0.0 | 0.0 | 0.0 | 0.0 | 0.0 | 0.0 | 0.0 | 0.0 | 0.0 | 0.0 | 0.0 |
| Furniture |  |  |  |  | 0.1 | 0.1 | 0.2 | 0.2 | 0.2 | 0.2 | 0.1 | 0.1 | 0.1 | 0.1 | 0.3 | 0.3 | 0.2 | 0.2 | 0.2 | 0.2 |
| Staff |  |  |  |  | 3.3 | 1.9 | 8.6 | 2.8 | 13.9 | 1.2 | 16.2 | 2.9 | 7.3 | 4.0 | 10.1 | 2.9 | 4.5 | 2.5 | 9.6 | 2.4 |
| Transport |  |  |  |  | 0.0 | 0.0 | 0.0 | 0.0 | 0.0 | 0.0 | 0.0 | 0.0 | 0.0 | 0.0 | 0.0 | 0.0 | 0.0 | 0.0 | 0.0 | 0.0 |
| Overheads |  |  |  |  | 0.3 | 0.3 | 0.3 | 0.3 | 0.5 | 0.5 | 0.5 | 0.5 | 0.3 | 0.3 | 1.7 | 1.7 | 2.3 | 2.3 | 0.8 | 0.8 |
| *SAT* | 125.4 | 93.2 | 264.5 | 91.6 | 39.8 | 18.4 | 33.6 | 27.8 | 30.5 | 8.1 | 31.9 | 9.1 | 28.3 | 21.8 | 18.4 | 13.0 | 23.0 | 19.6 | 21.1 | 9.2 |
| Building | 35.1 | 35.1 | 60.6 | 60.6 | 12.3 | 12.3 | 17.2 | 17.2 | 4.6 | 4.6 | 2.8 | 2.8 | 14.0 | 14.0 | 5.7 | 5.7 | 12.9 | 12.9 | 3.4 | 3.4 |
| Equipment | 1.7 | 0.0 | 4.4 | 0.0 | 0.1 | 0.0 | 0.0 | 0.0 | 0.1 | 0.0 | 0.0 | 0.0 | 0.1 | 0.0 | 0.0 | 0.0 | 0.0 | 0.0 | 0.0 | 0.0 |
| Furniture | 3.0 | 3.0 | 5.4 | 5.4 | 0.6 | 0.6 | 2.1 | 2.1 | 0.4 | 0.4 | 0.2 | 0.2 | 0.1 | 0.1 | 0.3 | 0.3 | 0.2 | 0.2 | 0.3 | 0.3 |
| Staff | 34.3 | 3.7 | 177.1 | 8.6 | 24.8 | 3.5 | 10.8 | 5.0 | 24.5 | 2.2 | 28.1 | 5.3 | 13.6 | 7.2 | 10.5 | 5.1 | 8.0 | 4.5 | 16.1 | 4.3 |
| Transport | 0.1 | 0.1 | 0.1 | 0.1 | 0.0 | 0.0 | 0.0 | 0.0 | 0.0 | 0.0 | 0.0 | 0.0 | 0.0 | 0.0 | 0.0 | 0.0 | 0.0 | 0.0 | 0.0 | 0.0 |
| Overheads | 51.2 | 51.2 | 16.9 | 16.9 | 2.1 | 2.1 | 3.5 | 3.5 | 0.9 | 0.9 | 0.9 | 0.9 | 0.5 | 0.5 | 1.8 | 1.8 | 2.0 | 2.0 | 1.3 | 1.3 |
| *Community-DOT* | 16.0 | 5.7 | 13.0 | 5.2 | ***-*** | ***-*** | ***-*** | ***-*** | ***-*** | ***-*** | ***-*** | ***-*** | ***-*** | ***-*** | ***-*** | ***-*** | ***-*** | ***-*** | ***-*** | ***-*** |
| Building | 2.1 | 2.1 | 0.3 | 0.3 |  |  |  |  |  |  |  |  |  |  |  |  |  |  |  |  |
| Equipment | 0.1 | 0.0 | 0.0 | 0.0 |  |  |  |  |  |  |  |  |  |  |  |  |  |  |  |  |
| Furniture | 0.2 | 0.2 | 0.0 | 0.0 |  |  |  |  |  |  |  |  |  |  |  |  |  |  |  |  |
| Staff | 10.6 | 0.4 | 12.6 | 4.8 |  |  |  |  |  |  |  |  |  |  |  |  |  |  |  |  |
| Transport | 0.0 | 0.0 | 0.0 | 0.0 |  |  |  |  |  |  |  |  |  |  |  |  |  |  |  |  |
| Overheads | 3.0 | 3.0 | 0.1 | 0.1 |  |  |  |  |  |  |  |  |  |  |  |  |  |  |  |  |

TD, top-down; BU, bottom-up; DOT, directly observed therapy; SAT, self-administered therapy; Transport refers to recurrent transport-related costs only.

## **Table S4 –** Potential savings: episode and total costs by type of treatment in the last two months of treatment for different utilisation assumptions (bottom-up estimates by site, USD 2013).

| Clinic number | 2 | 3 | 4 | 5 | 6 | 7 | 8 | 9 | | 10 | 11 |
| --- | --- | --- | --- | --- | --- | --- | --- | --- | --- | --- | --- |
| EPISODE COSTS BY TREATMENT TYPE | | | | | | | | | | | |
| Facility-DOT | | | | | | | | | | | |
| 45% continuation phase | - | - | 82.7 | 98.0 | 91.3 | 106.1 | 225.4 | 199.3 | | 373.3 | 108.8 |
| Patient utilisation | - | - | 83.5 | 99.1 | 92.3 | 107.2 | 228.0 | 201.5 | | 377.6 | 110.0 |
| SAT | | | | | | | | | | | |
| 45% continuation phase | 168.5 | 167.4 | 42.2 | 55.4 | 20.8 | 26.0 | 43.2 | 29.4 | | 41.0 | 22.8 |
| Patient utilisation | 187.6 | 186.2 | 46.0 | 61.1 | 22.4 | 27.9 | 47.6 | 32.1 | | 45.0 | 24.7 |
| Community-DOT | | | | | |  | | | | | |
| 45% continuation phase | 176.8 | 162.8 | - | - | - | - | - | | - | - | - |
| Patient utilisation | 185.6 | 170.8 | - | - | - | - | - | | - | - | - |
| TOTAL COSTS BY TREATMENT TYPE | | | | | | | | | | | |
| Facility-DOT | | | | | | | | | | | |
| 45% continuation phase | - | - | 9,010 | 6,372 | 4,931 | 848 | 18,260 | | 14,745 | 16,052 | 10,449 |
| Patient utilisation | - | - | 9,101 | 6,440 | 4,984 | 857 | 18,467 | | 14,910 | 16,234 | 10,562 |
| SAT | | | | | | | | | | | |
| 45% continuation phase | 505 | 334 | 337 | 55.4 | 914 | 467 | 2,202 | | 3,562 | 2,621 | 1,873 |
| Patient utilisation | 562 | 372 | 367 | 61.1 | 986 | 501 | 2,429 | | 3,884 | 2,877 | 2,028 |
| Community-DOT | | | | | | | | | | | |
| 45% continuation phase | 8,486 | 26,216 | - | - | - | - | - | | - | - | - |
| Patient utilisation | 8,907 | 27,500 | - | - | - | - | - | | - | - | - |
| TOTAL COSTS IN EACH FACILITY | | | | | | | | | | | |
| 45% continuation phase | 8,991 | 26,551 | 9,347 | 6,427 | 5,846 | 1,316 | 20,462 | | 18,307 | 18,673 | 12,322 |
| Patient utilisation | 9,470 | 27,873 | 9,469 | 6,501 | 5,971 | 1,358 | 20,896 | | 18,794 | 19,112 | 12,591 |

DOT, directly observed therapy; SAT, self-administered therapy.

Utilisation assumptions: 1) 45% continuation phase. In this scenario we calculated the costs in the last two months of treatment as a proportion of the continuation phase costs; 2) Patient utilisation. In this alternative scenario, costs in the last two months of treatment are calculated using the reported number of visits by patients interviewed.

## **Table S5 –** Potential savings: episode and total costs by type of treatment in the last two months of treatment for different utilisation assumptions (top-down estimates by site, USD 2013).

| **Clinic number** | **2** | **3** | **4** | **5** | **6** | **7** | **8** | **9** | **10** | **11** |
| --- | --- | --- | --- | --- | --- | --- | --- | --- | --- | --- |
| **EPISODE COSTS BY TREATMENT TYPE** | | | | | | | | | | |
| Facility-DOT | | | | | | | | | | |
| 45% continuation phase | - | - | 108.5 | 205.3 | 328.4 | 353.0 | 288.1 | 332.9 | 410.5 | 243.1 |
| Patient utilisation | - | - | 109.6 | 207.6 | 332.1 | 357.0 | 291.4 | 336.6 | 415.2 | 245.8 |
| SAT | | | | | | | | | | |
| 45% continuation phase | 225.0 | 470.8 | 79.7 | 65.6 | 60.1 | 65.9 | 54.6 | 38.9 | 47.0 | 43.6 |
| Patient utilisation | 250.7 | 525.0 | 87.9 | 72.5 | 66.3 | 72.4 | 60.3 | 42.6 | 51.7 | 47.9 |
| Community-DOT | | | | | | | | | | |
| 45% continuation phase | 485.6 | 397.6 | - | - | - | - | - | - | - | - |
| Patient utilisation | 510.1 | 417.6 | - | - | - | - | - | - | - | - |
| **TOTAL COSTS BY TREATMENT TYPE** | | | | | | | | | | |
| Facility-DOT | | | | | | | | | | |
| 45% continuation phase | - | - | 11,823 | 13,343 | 17,732 | 2,824 | 23,338 | 24,631 | 17,653 | 23,333 |
| Patient utilisation | - | - | 11,947 | 13,492 | 17,933 | 2,855 | 23,603 | 24,911 | 17,854 | 23,595 |
| SAT | | | | | | | | | | |
| 45% continuation phase | 675 | 941 | 637 | 65.6 | 2,643 | 1,186 | 2,782 | 4,701 | 3,009 | 3,574 |
| Patient utilisation | 752 | 1,050 | 703 | 72.5 | 2,918 | 1,304 | 3,077 | 5,157 | 3,311 | 3,928 |
| Community-DOT | | | | | | | | | | |
| 45% continuation phase | 23,308 | 64,012 | - | - | - | - | - | - | - | - |
| Patient utilisation | 24,486 | 67,226 | - | - | - | - | - | - | - | - |
| **TOTAL COSTS IN EACH FACILITY** | | | | | | | | | | |
| 45% continuation phase | 23,983 | 64,954 | 12,461 | 13,408 | 20,375 | 4,010 | 26,121 | 29,333 | 20,663 | 26,907 |
| Patient utilisation | 25,238 | 68,276 | 12,650 | 13,565 | 20,851 | 4,159 | 26,681 | 30,068 | 21,166 | 27,524 |

DOT, directly observed therapy; SAT, self-administered therapy.

Utilisation assumptions: 1) 45% continuation phase. In this scenario we calculated the costs in the last two months of treatment as a proportion of the continuation phase costs; 2) Patient utilisation. In this alternative scenario, costs in the last two months of treatment are calculated using the reported number of visits by patients interviewed.

## **Table S6 –** Demographic and clinical characteristics of TB patients interviewed during the last two months of first-line treatment in Rio de Janeiro, Brazil.

| **Variables** | **N** | **%** |
| --- | --- | --- |
| **Sex** |  |  |
| Female | 49 | 39 |
| Male | 77 | 61 |
| **Age,** median (IQR) | 126 | 43.0 (27.8-55.4) |
| **Tuberculosis site** |  |  |
| Pulmonary | 111 | 88 |
| Extra pulmonary | 14 | 11 |
| Both | 1 | 0 |
| **HIV infection status,** positive | 9 | 7 |
| **Smear results** |  |  |
| Positive | 74 | 59 |
| Negative | 32 | 25 |
| Not done | 20 | 16 |
| **Treatment history** |  |  |
| New case | 102 | 81 |
| Retreatment | 24 | 19 |
| *Relapse* | *15* | *63* |
| *Failure* | *3* | *25* |
| *Default* | *6* | *12* |
| **Treatment Regimen** |  |  |
| 2RHZE/4RH, FDC | 122 | 97 |
| Other | 4 | 3 |
| **Residency** |  |  |
| Urban | 86 | 68 |
| Peri-urban | 40 | 32 |
| **Type of therapy** |  |  |
| Facility-DOT | 47 | 37 |
| SAT | 63 | 50 |
| Community-DOT | 16 | 13 |
| **Number of visits in the last 2 months,** mean (SD) | | |
| Facility-DOT | 47 | 18.8 (13.1) |
| SAT | 63 | 1.96 (0.4) |
| Community-DOT | 16 | 31.5 (14.2) |
| **Monthly income,** median (IQR) | 49 | 370 (298-625) |
| **Socioeconomic status** |  |  |
| A1 /A2 (USD 4,288.4) | 3 | 3 |
| B1 (USD 2,426.3) | 4 | 3 |
| B2 (USD 1,228.7) | 19 | 16 |
| C1 (USD 780.1) | 39 | 33 |
| C2 (USD 531.0) | 37 | 31 |
| D/E (USD 359.2) | 17 | 14 |

IQR; interquartile range; RHZE, rifampicin, isoniazid, pyrazinamide, ethambutol; FDC, fixed-dose combination; SAT, self-administered therapy; DOT, directly observed therapy; SD, standard deviation.

## **Table S7** - Proportion of total costs over income, according to different methods to estimate income.

|  | **Minimum wage, median (IQR)** | **SES income per capita, median (IQR)** | **Income per activity, median (IQR)** | **Reported income, median (IQR)*** |
| --- | --- | --- | --- | --- |
| **Overall** | 26.3% (1.6%-41.8%) | 10.4% (1.4%- 41.8%) | 21.5% (5.6%-54.2%) | 12.8% (3.2%; 54.3%) |
| **Type of therapy** |  |  |  |  |
| Facility-based DOT | 29.9% (11.6%-76.4%) | 12.2% (4.3%-44.9%) | 25.1% (12.4-57.7%) | 28.2% (9.4%-37.8%) |
| SAT | 34.5 % (4.3%-93.0%) | 11.1% (1.2%-48.7%) | 22.2% (4.5%-52.4%) | 6.2% (3.0%; 53.4%) |
| Community-based DOT | 3.6% (2.3%-40.2%) | 1.6% (0.7%-14.0%) | 4.0% (2.5%-20.5%) | 7.8% (3.0%; 53.4%) |
| **Catastrophic costs** |  |  |  |  |
| Yes | 56 (44%) | 34 (27%) | 43 (34%) | 19 (39%) |
| No | 70 (56%) | 92 (73%) | 83 (66%) | 30 (61%) |

SAT, self-administered therapy; DOT, directly observed therapy; IQR, interquartile range.

* Among 49 patients, only 42 declared income higher than zero.

## References

1. Sinanovic E, Kumaranayake L. Quality of tuberculosis care provided in different models of public-private partnerships in South Africa. Int J Tuberc Lung Dis. 2006 Jul;10(7):795–801.

2. Sinanovic E, Floyd K, Dudley L, Azevedo V, Grant R, Maher D. Cost and cost-effectiveness of community-based care for tuberculosis in Cape Town, South Africa. Int J Tuberc Lung Dis. 2003 Sep;7(9 Suppl 1):S56–62.

3. Costa JG, Santos AC, Rodrigues LC, Barreto ML, Roberts JA. [Tuberculosis in Salvador, Brazil: costs to health system and families]. Rev Saude Publica. 2005 Feb;39(1):122–8.

4. Prefeitura do Rio de Janeiro. data.rio [Internet]. [cited 2012 Nov 11]. Available from: http://data.rio.rj.gov.br/

5. CNESNet. Cadastro Nacional de Estabelecimentos de Saúde - CNES [Internet]. [cited 2012 Nov 11]. Available from: http://cnes.datasus.gov.br/

6. The World Bank. Official exchange rate (LCU per US$, period average) [Internet]. Available from: http://data.worldbank.org/indicator/PA.NUS.FCRF
